# Healthy, safe and effective international medical student electives: a systematic review and recommendations for program coordinators

**DOI:** 10.1186/s40794-019-0081-0

**Published:** 2019-04-03

**Authors:** D. Ashley Watson, Nicholas Cooling, Ian J. Woolley

**Affiliations:** 1 Australian National University Medical School - Canberra Hospital Campus, Garran, ACT Australia; 2 School of Medicine, University of Tasmania, Tasmania, Australia; 3 Monash Infectious Diseases, Monash Health and Monash University, Clayton, Victoria Australia

**Keywords:** Global, Ethics, Travel, Occupational, Risk

## Abstract

**Background:**

Thousands of medical students undertake international medical electives each year. These students face potentially substantial health and safety risks as well as educational and ethical challenges and therefore should undertake their electives within well-structured and coordinated programs.

**Methods:**

We conducted a qualitative systematic review based on a pre-determined protocol. Relevant publications and guidelines relating to international medical electives were identified through a review of the literature using on-line search engines, principally PubMed. We then conducted a systematic qualitative synthesis to extract relevant publications. Finally, the literature was organized according to themes, with the aim of developing a structured set of Recommendations for Implementation for program coordinators.

**Results:**

A wide range of important issues were identified which were categorized into seven themes upon which recommendations were made principally for the benefit of program coordinators: Responsibilities; General policies; Travel advisories; Occupational risk assessment; Funding & finances, Pre-departure training programs; and Post-return debriefing and screening.

**Conclusions:**

Recommendations for program coordinators on the health and safety of medical students while on international medical electives have been sourced from existing guidelines and relevant publications. There was considerable consensus from the literature and as such these synthesised recommendations could form the basis for internationally accepted standards for elective placement program coordinators.

**Electronic supplementary material:**

The online version of this article (10.1186/s40794-019-0081-0) contains supplementary material, which is available to authorized users.

## Introduction

A significant proportion of medical students from high-income countries around the world participate in enriching, usually self organised, clinical electives outside their home country - often known as international medical electives or global health electives [1]. This is now also becoming increasingly popular in low- and middle-income countries - although the motivations of students from different regions may be dissimilar [2]. Electives typically last between one and three months, with some students undertaking sequential placements in two or more locations. International medical electives are generally clinical immersion experiences, with student contributions ranging from passive observation to active involvement in multiple aspects of care, including clinical assessment, case management, and participation in invasive procedures – all occurring under widely varying degrees of supervision. In addition to the clinical placements themselves, many students add cultural side trips and wilderness or other adventures to their itineraries. Although many of these electives are organized by the students themselves, an increasing number are arranged through faculty partnerships, philanthropic sponsorships, and commercial volunteer placement organizations [3, 4].

International medical electives tend to be of higher risk than clinical placements at home. While that risk may be due to increased exposure to infectious diseases and limited access to safety equipment, overall clinical activities are usually a lesser threat than the more general aspects of travel, including road travel, adventure activities, casual sex, and recreational drug use [5]. Whilst standard pre-travel health advice covers some of the issues relevant to electives, there are typically a number of additional issues that need to be considered, including occupational hazards, professional standards, emotional wellbeing, and ethical challenges. Certain locations and contexts are of higher risk and as such health advice may need to be tailored to those situations. The health and medical history of individual students also need to be taken into account.

In contrast, universities are becoming more risk averse about students on international placements and those concerns include potential harms to their students and reputational risk to the institution [6]. Threats are perceived to be increasing associated with climate related extreme weather events, terrorism, political unrest and infectious disease vector spread [7]. There is also increasing awareness of the burden and ethical harm placed on some developing country host placements [8, 9].

The aim of this review is to determine the current best practice in preparing medical students for international electives. The recommendations would be of particular benefit to international elective program coordinators in developing structured programs with local guidelines to ensure that their students stay physically and emotionally healthy and safe while engaging in clinical and non-clinical experiences that should be enriching to students and mutually beneficial to students and their host institutions.

## Methods

There were three parts to our methodology: a literature review, a systematic qualitative synthesis and finally the generation of recommendations. Together they form a qualitative systematic review based on a pre-determined protocol. This process aimed to encourage integrity and trustworthiness of results, consistency between reviewers, and ensured data extraction and synthesis were not arbitrary [10].

**Fig. 1:**
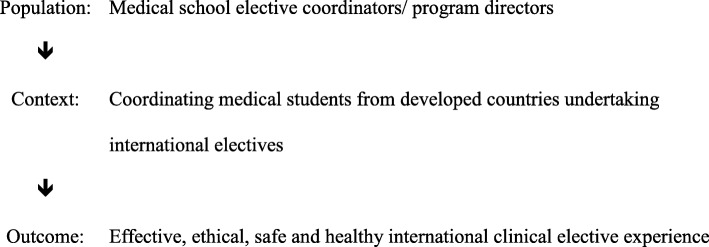
Component definition of the research question [79]

Our research question was: what is the best practice for elective program coordinators to ensure effective medical student electives in international settings? This question can be defined according to population, context and outcome as shown in Fig. 1 below.

### Literature review

A review of the literature, including the grey literature and publications and guidelines relating to international medical electives in low- and middle-income countries, was undertaken through an advanced search by titles of PubMed [11] and Google Scholar [12] using the search strategy: (“international” OR “global” OR “developing country” OR “low resource country” OR “low income country” OR “middle income country”) AND (“medical” OR “health”) AND (“electives” OR “experiences” OR “placements”). We restricted our search to articles that were peer reviewed, written in English and published between 2000 and 2018.

The search yielded 1479 titles (121 from the Google Scholar database and 1358 from PubMed) and after removal of duplicates and screening for eligibility, 101 full text publications were extracted.

Eligible publications were then separated into ‘comprehensive guidelines’ (Table 1) and other ‘supportive literature’. Since almost all comprehensive guideline publications reviewed were rated as low level evidence, based on expert opinion there was no attempt to stratify the publications according to level of evidence used in quantitative systematic reviews. However, the comprehensive guidelines were nevertheless critiqued for strengths and weaknesses. In addition, we used a Text and Expert Opinion Critical Appraisal Tool devised by McArthur et al. to assess their quality (Additional file 1) [13]. We did not review the quality of the selected ‘other supportive’ literature.

**Table 1:** Existing guidelines relevant to international medical electives

Guideline	Region	Year	Text & Expert Opinion CAT score [14]	Comment
AFMC Global Health Resource Group: Preparing medical students for electives in low resource settings: a template for national guidelines for pre-departure training [45]	Canada	2008	6	Grey literature; 10 years old; Comprehensive but does not separate faculty & student recommendations & responsibilities.
WEIGHT: Ethics and best practice guidelines for training experiences in global health [41]	International	2010	6	Very comprehensive; Expert working group; Not specifically aimed at medical student electives or program coordinators.
General Medical Council: Clinical placements for medical students [80]	UK	2011	6	Grey literature; Generic document for all clinical placements.
A guide to working abroad for medical students and junior doctors [70]	Australia	2011	5	Very broad and approachable document written by the lead doctors and students organisations of Australia. Uses many casestudies and largely focussed on humanitarian and service electives.
Guidelines for safety of trainees rotating abroad: consensus recommendations from the Global Emergency Medicine Academy of the Society for Academic Emergency Medicine, Council of Emergency Medicine, Council of Emergency Medicine Residency Directors, and the Emergency Medicine Residents’ Association [81]	U.S.A.	2013	6	Comprehensive; Aimed at emergency medicine electives (students & residents); Emphasis on personal safety.
AMEE Guide No. 88: Electives in undergraduate medical education [49]	UK	2014	6	Comprehensive review; Easily readable narrative & examples; Does not separate faculty & student recommendations.
Australian Outbound Student Mobility: Quality dimensions for international fieldwork in health sciences [67]	Australia	2015	5	Grey literature; very broad-based - aimed at general higher-education audience; Many good-practice examples.
WMA Statement on Ethical Considerations in Global Medical Electives [19]	International	2016	5	Grey literature; Short statement on key ethical issues.
Preparing for International Health Experiences: a practical guide [82]	North America	2017	5	Commercially published book; Aimed at travelling healthcare workers; Not written specifically for program coordinators.
Global Health: Preparation for Working in Resource-Limited Settings [20]	U.S.A.	2017	5	Comprehensive thematic approach; Not specifically aimed at program coordinators.
Recommendations for undergraduate medical electives: a UK consensus statement [83]	U.K.	2018	6	Aimed at all types of electives, including local placements; Comprehensive, although little detail on health & safety risks.
Working in global health: A planning and implementation framework for international electives [84]	Australia	2018	5	Written by two medical students and an academic supervisor; Provides a checklist of recommendations with an emphasis on social accountability.

### Systematic qualitative synthesis

Data extraction was be undertaken by one author (DAW). The following information, where possible, was extracted from each ‘comprehensive guideline’ article: Bibliographic information; study aims; study design: methodological underpinnings; inclusion/exclusion criteria; data collection on methods; data analysis techniques; results: themes, quotes, author interpretations or explanations; strengths and limitations; and any reviewer comments.

The extracted data was then analysed utilising thematic analysis techniques, allowing clear identification of themes arising from the data, and a metasynthesis process outlined by Thomas and Harden (2008) [1] by one author (DAW). This systematic qualitative (meta) synthesis process included brief coding of the findings contained in the ‘comprehensive guideline’ publications. Data was then examined for meaning and content during the coding. This process allowed the translation of codes and concepts between studies. The codes were then analysed for their meanings, and reorganized into related categories. Each category was then compared to other categories, specifically looking for similarities and differences. Similar categories were merged into themes. All authors then reviewed the list of generated themes and made suggestions for additional, or amalgamation of, themes. After this process was conducted on the 12 comprehensive guideline publications, the other 89 supportive literature documents were briefly reviewed by all authors and if additional themes were identified they were added to the list.

### Development of recommendations

Under each of the themes key publications were allocated and from these publications, key recommendations developed. This process was initially performed by the lead author (DAW) and then checked and refined by the other two authors.

Both the existing guidelines and supportive literature formed the basis for generating specific recommendations for, a) elective program coordinators (Table 2); b) medical students (Table 3); and c) travel doctors (Table 4).

**Table 2:** Recommendations for implementation by medical student elective program coordinators

Issue	Recommendation	Reference
Responsibilities	Determine those issues that are the responsibility of the Medical School and distinguish from those that are the responsibilities of students. Inform students of their responsibilities (Table 3).	[3, 24, 27, 42, 49]
	Consider creating a detailed webpage for the program, to be kept up-to-date and embedded with an institutional site.	[15, 16]
	Assess all elective applications on the basis of personal risk and educational merit.	[27, 49]
	Develop a range of enduring elective destinations including the establishment of strong and mutually beneficial partnerships with international organizations, universities and health care providers.	[1, 85]
	Site visits to host institutions to provide quality assurance in academic standards and ethics, based on context and partnership agreements	[1, 22, 73]
General Policies	Provide students with policies on electives, including choices of destination country and clinical experiences.	[32, 41, 49]
	Provide students with guidelines on health and safety on electives.	[21, 27, 32, 41]
	Inform students of medical indemnity, public liability and personal protection insurance provisions for electives.	[27]
	Policies should be created in collaboration with host institutions, particularly where formal partnerships are being forged	[24]
	Policies need to be implemented and a degree of compliance achieved by both elective program coordinators and students.	[85]
Travel advisories	Consider elective destinations in the light of national travel advisories and be prepared to deal with unexpected changes in risk status.	[24, 32]
	Ensure that students travelling to higher-risk destinations have sought pre-travel health advice and have purchased travel insurance.	[5, 21, 32, 49]
	Instruct students to register with institutional travel registries.	[33, 34]
	Provide students with 24-h faculty contact for urgent issues.	[32, 49]
Occupational risk assessment	Consider providing travel doctors with information on occupational risk management in electives.	[32, 41, 49]
	Establish policies on HIV PEP, including supply and funding.	[49]
Pre-departure training programs	Ensure that pre-departure training is provided for students, covering educational outcomes, clinical experiences, ethics and social accountability, occupational risk management, health and safety.	[32, 41, 42, 44, 47, 56, 59, 60, 64, 86]
	Reinforce learning from training modules with pertinent and enlightening reading material from peer-reviewed literature on international medical electives.	[8, 9, 23, 49, 55, 59, 87]
	Ensure that experienced faculty play a central role in planning and implementing training sessions to ensure quality and sustainability.	[32, 47, 60]
	Include procedural skills in pre-departure training.	[38, 88]
Funding and finances	Ensure that students are aware of the approximate costs of electives, including the need to budget for travel insurance and vaccinations.	[41, 59]
	Inform students of bursaries, scholarships and other funding support that may be available.	[49]
	Sending organisations and students should consider funding host institutions to compensate for resources used during the placement and to develop the host institution	[54]
Post-return debriefing and screening	Ensure that students are offered post-elective debriefing sessions. Consider providing travel doctors with information on post-return health screening.	[86]
	Encourage students to reflect on their elective experience through reflective writing, discussion groups or academic debriefing.	[23, 47, 49, 63]
	Seek feedback from host institutions on both individual students and broader programmatic issues.	[9, 49]

**Table 3:** Recommendations for implementation by students undertaking medical electives

Issue	Responsibility	Reference
General	Participate in pre-departure training and post-return debriefing programs	[42, 47, 56, 59, 86]
Funding and Finances	Ensure adequate funds for airfares, accommodation, comprehensive travel insurance, daily living expenses, vaccinations and medications, visas, placement fees, recreational activities.	[4]
	Establish the means by which HIV post-exposure prophylaxis (if required) will be prescribed and funded.	[22, 23, 27]
Clinical Routine	Establish contact with the designated supervisor and ascertain details of the elective program, clinical experience required and level of supervision.	[59, 63, 69]
	Be aware of skills gaps that may be addressed prior to departure and that should be acknowledged as such during the elective.	[69, 70]
	Become familiar with syndromic approaches to management of common and important conditions in host country.	[65, 89]
Accommodation and Personal Safety	Consider undertaking the elective with another student.	[27, 39]
	Confirm the availability of secure and screened accommodation; determine whether guards and/or domestic help are provided.	[49]
	Be streetwise when ‘out and about’; seek advice from hosts and inform supervisors of local travel plans. Undertake sufficient planning for and exercise caution when travelling by vehicle; avoid riding motorcycles.	[6, 23, 28]
	Uphold high standards of clinical practice; seek advice and support from colleagues when dealing with ‘difficult’ patients.	[70]
	Register with institutional and government travel registries prior to departure.	[29, 30, 33, 34, 74]
Ethical Behaviour and Social Accountability	Perceive the elective experience as that of a privileged visitor in a welcoming host community, value clinical cultures, respect social norms, work on language skills, and adapt attitudes and behaviour accordingly.	[17, 18, 23, 59, 76]
	Dress modestly and respectfully; observe standards of clinical attire.	[23]
	Undertake an institutional and/or on-line training program in global health ethics.	[64]
Health and Wellbeing	Ensure that adequate pre-travel health and safety advice, including necessary vaccines and medications, and appropriate preventative behaviour education (i.e. mosquito avoidance, safe sex) is obtained in a timely manner from a travel doctor. Pack a supply of non-sterile gloves, goggles and N-95 masks. Ensure that chronic medical conditions (including mental health conditions) are optimally controlled and that a management plan is in place for exacerbations. Arrange a pre-travel dental check-up.	[23, 25, 78]
	Be competent in the self-management of minor illnesses and injuries, as well as recognition of symptoms of potentially serious illnesses.	[6]
	Know how and where to access affordable local medical care, including services aimed specifically at foreigners. Keep all receipts and promptly notify travel insurance providers of all incidents and consultations.	[49]
	In the event of significant illness or injury, seek the support and companionship of another student; notify the program coordinator or other faculty member.	[49]

**Table 4:** Recommendations for travel doctors undertaking occupational risk management for medical electives

Issue	Recommendation	Reference
General	Advise students that occupational risks are accentuated by various factors, including greater demands and opportunities to perform invasive procedures, poorly resourced work areas, lack of personal protective equipment, fatigue from activities, and often greater caseloads compared with prior experiences in home institutions.	[90]
Tuberculosis	Inform students that tuberculosis (TB) is common in lower income countries, particularly in sub-Saharan Africa, Papua New Guinea, and the poorer regions of Asia and South America. Multidrug-resistant tuberculosis is a notable emerging threat. Medical students are commonly exposed to TB while on their medical electives.	[36, 91–94]
	Educate students on modes of transmission of TB and minimisation of exposure risk, particularly through the wearing of high-filtration masks. Enlighten students about potential obstacles to risk avoidance, including lack of adequately ventilated rooms, delays in suspected case identification, limited diagnostic facilities, reluctance to wear masks, limited resources for directly observed therapy, and reliance on passive case finding.	[36, 95, 96]
	Undertake baseline testing for latent TB infection prior to departure (interferon gamma release assay or tuberculin skin testing).	[97]
	Consider BCG vaccination in students not previously vaccinated, especially if working in TB wards in countries with high rates of multidrug or extended-drug-resistant TB.	[98]
	Recommend follow-up testing for TB infection 8–12 weeks after return. The object of testing is to identify and treat students who have been recently infected and who are thus at significant risk of progression to clinical disease.	[36]
Blood-Borne Viruses	Ensure that students are aware that blood-borne viruses are common in many elective destinations and that occupational exposures occur in elective students. Students should be aware of their own HIV, HBV and HCV serological status (including proven immunity to hepatitis B) and should know the principles of risk management when performing invasive procedures.	[99–102]
	Expect all sharps injuries to be managed according to guidelines, including cleansing of the wound, incident reporting and documenting, baseline testing of source patients and exposed persons, consideration of post-exposure prophylaxis (PEP) and follow-up serological testing.	[103]
	Ensure that PEP for HIV infection is available to students where the risk of occupational exposure is significant. Students must be prepared to make quick decisions regarding empiric commencement of PEP – ideally within two hours of any incident. Consider PrEP for higher risk situations.	[101, 104, 105]
	Ensure that students have been vaccinated against hepatitis B unless naturally immune or already chronically infected. Students who are uncertain about their hepatitis B serostatus should be tested.	[106, 107]
Personal violence	Ensure that students are aware of the risk of personal violence in clinical settings. Advise students to remain vigilant at all times for the warning signs of patient distress and aggressive behaviour. Inform students that they must withdraw from potentially violent situations and seek immediate advice and support from colleagues and supervisors.	[28, 108]
Other Diseases	Given the typically short duration and high stakes nature of electives, encourage a high degree of vigilance to minimize the risk of food and water-borne diseases.	[109]
	Recommend vaccination against hepatitis A unless vaccinated or proven to be immune from natural infection.	[107]
	Consider vaccination against typhoid fever, the actual risk of infection varying somewhat from region to region.	[107]
	Consider cholera vaccination if travelling to areas affected by humanitarian crises or notable high-risk regions. The vaccine may also afford cross-protection against traveller’s diarrhoea due to enterotoxigenic *Escherichia coli*.	[107]
	Ensure up-to-date vaccination against influenza, varicella, diphtheria, tetanus, pertussis, measles, mumps, rubella and poliomyelitis.	[110–112]
	Consider vaccination against meningococcal disease, owing to potential exposure to clinical cases and likely exposure to new serotypes in the general community. Essential for students travelling to the ‘meningitis belt’ of equatorial Africa.	[112, 113]
	Ensure guidelines for prevention of other exotic diseases are followed, including those for arboviral and other mosquito-borne (particularly yellow fever, Ebola, dengue, Zika and malaria) and rabies.	[107]
Emotional wellbeing	Recognize risk factors for emotional distress include travelling alone, prior mental illness, low levels of resilience, insufficient pre-departure training and perceived or actual lack of support during the elective. Adequate preparation, in-country support and post-return debriefing are likely to reduce emotional distress arising from situations experienced on an elective.	[47, 58]

## Results

### Literature review

Twelve existing guidelines relevant to international medical electives were identified and summarised (Table 1). These existing guidelines were mostly written by experts and consensus panels and derived from USA, Europe and Australia/New Zealand. They were critiqued for strengths and weaknesses as indicated in the comment column and scored for quality using the Text and Expert Opinion Critical Appraisal Tool. They all met sufficient levels of quality to be included in this review.

The 12 comprehensive guideline papers and the 89 supporting literature publications were subjected to a systematic qualitative synthesis process.

### Systematic qualitative synthesis

Through systemic qualitative synthesis 11 themes emerged, with seven themes being directly relevant to program coordinators: Responsibilities; General policies; Travel advisories; Occupational risk assessment; Pre-departure training programs; Finances & Funding and Post-return debriefing and screening. This was the intended main focus of this study.

However it became clear that many of the emerging themes also related to students’ interests. We found six themes related to students, two of them overlapping with program coordinator interests (‘general’ and ‘funding & finances’) with four of them being more relevant to students than program coordinators: clinical routine, accommodation and safety, ethics, and health and safety. Nevertheless program coordinators have an interest in all 11 themes, if only to be aware on how they impact on the student experience.

Whilst we felt that recommendations for travel doctors needed to also be included, these were based on critical disease and illness states likely in international electives rather than on themes (Table 4). Detailed analyses of the themes are outlined below.

### Theme 1. Responsibilities

The responsibility for the undertaking and outcome of a safe and beneficial international medical elective is principally shared between a medical school and individual students. However, it is ultimately the sending institution that bears the responsibility for safety, health and effective learning – as in any clinical placement. Medical schools (being ‘sending’ or ‘sponsor’ institutions) and their delegated elective program coordinators are typically responsible for programmatic issues such as indemnity, travel advisories, faculty and student travel registries, general policies curriculum requirements, pre-departure training programs and ‘distant support’ while in country. Embedding the structure and up-to-date details of an established medical electives program in the institutional website should be considered, highlighting mission statements, goals and partnerships, as well as promoting the program locally and internationally [14–16].

Student responsibilities for electives are far more varied and apply to the details of applications, health and safety, clinical activities, ethical behaviour, and so on. Although program coordinators cannot oversee or be held accountable for every aspect of the many parts that ultimately lead to a successful elective, they do have an obligation to ensure that students planning international medical electives are made aware of their responsibilities (Table 3) [17–19]. For example, some form of audit of compliance with key tasks may be required.

In addition, there are responsibilities for travel doctors who may be consulted both before and after an elective by the medical student. Usually these travel consultations are encouraged or even mandated by the program coordinator, but organised by the student. They usually involve screening and advice about certain ‘occupationally related’ infectious diseases or injuries (Table 4).

**Fig. 2:**
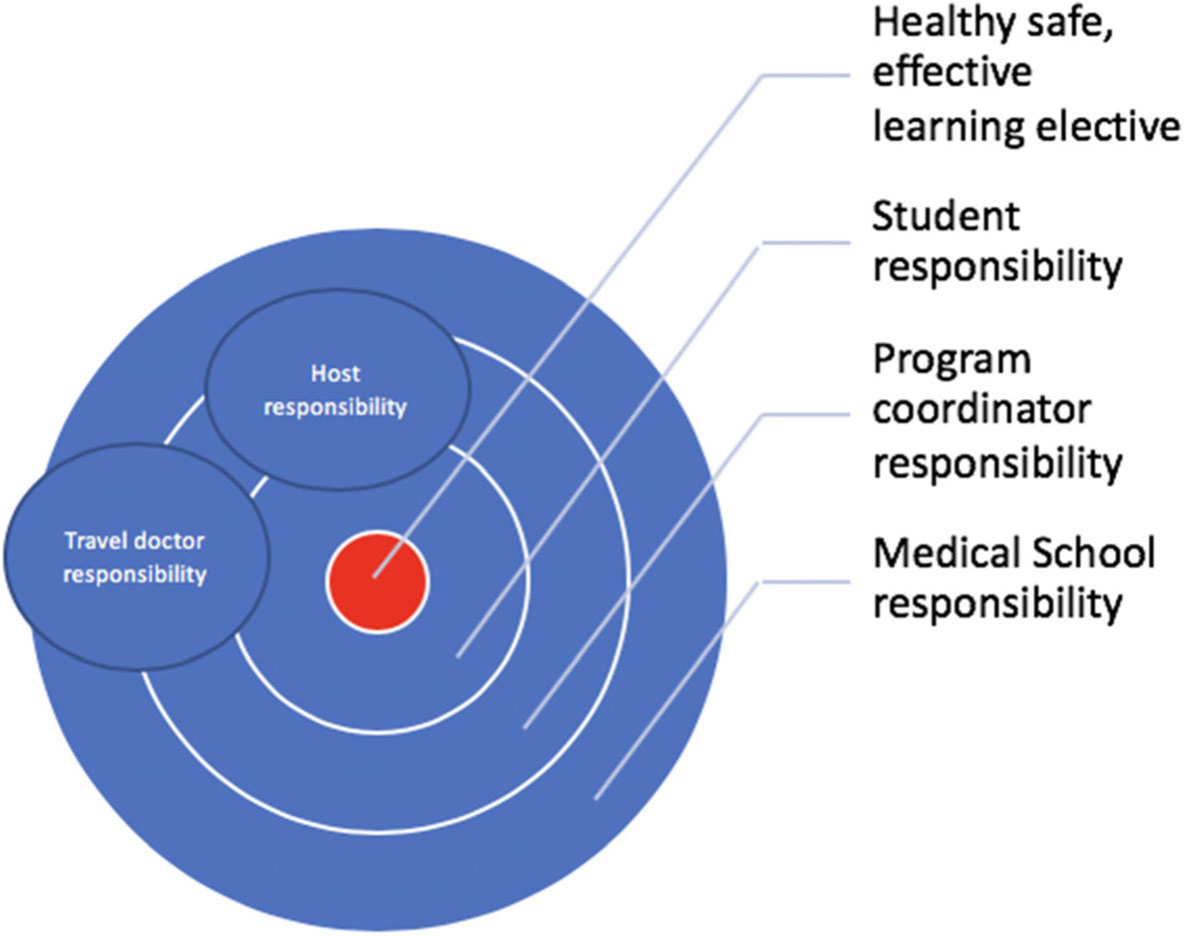
Shared responsibilities to achieving a safe, healthy and effective learning elective

Finally, the host institution also bears some responsibility and this is increasingly so with institutional partnerships and two-way elective exchanges. Formal agreements (e.g. work-integrated learning contracts) can be used to lay out the responsibilities of both the sending and host institution (principally clinical supervision) during a medical elective. Increasingly medical schools are sending academics and other officials to host institutions, often while students are on elective placement, to directly experience the clinical setting including supervision, and guide its context and relationship to the home curriculum. The other drivers for site visits are risk management, quality assuring technical expectations, ethical considerations and the intention of building enduring partnerships [2, 20, 21]. These site visits also provide the opportunity for elective coordinators to develop longitudinal projects relevant to medical students, jointly agreed upon by the home institution in consultation with the host community [22]. The sending organisation also has an obligation to evaluate the outcomes - positive and negative - and work with the host to optimize the benefit and reduce any harm from the elective [23]. The intertwining of these responsibilities from various parties is summarised in Fig. 2.

### Theme 2. General policies

The extent to which sending institutions are involved in the organization and supervision of electives ranges from programs where students themselves organize just about everything, to robust and well-funded global health partnerships between medical schools and hospitals in high and low- and middle-income countries, respectively [24, 25]. Whatever the model, written and regularly updated policies and guidelines should be in place, and readily available to students. Ideally, these policies should be created in collaboration with host institutions, particularly where formal partnerships are being forged [19]. These policies will likely complement broader policies on infectious diseases and professional standards that apply to the whole medical school experience. Policies need to be implemented and a degree of compliance achieved by both elective program coordinators and students.

Medical schools have a legal duty of care towards both students and patients [19, 26]. This duty of care extends to medical electives and includes the entire elective experience and the risks and hazards that may be encountered [25]. Many universities provide professional indemnity, public liability, and travel and personal protection insurance for their staff and students, which, for medical students, should apply to electives in most countries. Some medical schools expect students to also take out their own medical indemnity insurance (often provided free to students by medical defence organizations). Host elective institutions often also require proof of medical indemnity insurance.

### Theme 3. Travel advisories

Medical schools typically focus on the broader hazards of destination countries when analysing risk on electives. Most universities already have established rules about travel to high-risk destinations that apply to all staff and students and compliance with these rules and corresponding institutional risk management systems is generally desirable [27]. For travelling students, the threat of civil unrest, crime and personal safety in a chosen country is somewhat unpredictable but may be reduced by choosing lower-risk elective locations and by taking a few common-sense precautions from the time of arrival at the chosen destination. Trauma from motor vehicle crashes, recreational activities and personal violence is probably the greatest risk faced by students and perhaps the most difficult to mitigate [28]. Many countries provide travel advisories and registries for their citizens and one approach for medical schools is to restrict electives to the safer categories of travel warnings [29–31]. Students and their program coordinators should also be prepared for late, sudden changes in security conditions, and the emergence of epidemic and climatic threats in their intended destinations [32]. Notwithstanding any reasonable restrictions that medical schools may place on choices of electives and additional travel, program coordinators should keep in mind that a balance does need to be achieved between restrictive policies and students’ rights to make informed choices – many students are already seasoned travellers by the time they start planning their electives.

Program coordinators should also ensure that their medical schools maintain a readily accessible on-line record of each elective student’s intended itinerary, placement particulars and contact details. This should also include contact information for a student’s next-of-kin or other responsible person in the home country. Many universities now have official faculty and student travel registries (resembling government-endorsed travel registries) that should not only facilitate the process of tracking students but also ensure sufficient compliance with broader institutional rules [33, 34].

### Theme 4. Occupational risk assessment

There are a number of important clinical occupational hazards on medical electives that require due consideration by both program coordinators and students. These include pulmonary tuberculosis and other respiratory infections, human immunodeficiency virus (HIV) and other blood-borne viruses, threats of personal violence from aggrieved, psychotic or delirious patients, and the broader dangers, fear and uncertainty that arise during humanitarian crises. The threat of tuberculosis is worsened by the emergence of multidrug resistance in many countries [35]. Moreover, patients in low-income countries commonly present with advanced disease and clinical diagnoses are frequently delayed – undiagnosed patients often posing a greater threat than diagnosed cases. Students need to have an understanding of the pathogenesis and transmission of tuberculosis and be informed of measures that should be undertaken to minimise the risk of exposure [36].

Some medical schools discourage electives in certain locations on the basis of occupational risk, particularly in relation to participation in invasive procedures in settings where there is a high prevalence of HIV infection. For example, electives in trauma, surgery, and obstetrics in sub-Saharan African countries may be entirely prohibited [37]. Other factors need to be taken into account, however, such as the extent to which students will undertake or assist in exposure-prone procedures, and the degree of supervision and quality of risk management that is believed to exist in the clinical environment. Program coordinators should therefore perhaps assess student elective proposals carefully and individually, rather than applying more sweeping restrictions. Interventions that may reduce the risk of clinical exposure to HIV infection include the provision of pre-departure training programs, procedural skills competency training, and the prescribing – sometimes including provision – of antiretroviral post-exposure prophylaxis (PEP) [38]. Procedural skills training generally should differ little from the training students already receive in their home curricula, the main qualification being an awareness of disparities in available resources, including the accessibility and reuse of disposable equipment [8]. The increasing availability of and coverage with antiretroviral therapy in lower and middle-income countries should also reduce the risk of HIV transmission through clinical procedures. Some host institutions make PEP routinely available to elective students as well as their own staff, while others may suggest bringing a PEP combination from their home country [39]. Generic PEP can now be obtained relatively cheaply through online providers on submission of a valid prescription [40].

With the advent of effective antiretroviral pre-exposure prophylaxis (PrEP) medical students should also be made aware of the protocol and to its side effects and benefits. As with PEP, an awareness of the HIV prevalence in the elective location and the determination of the exposure risk, will guide its use.

The threat of personal violence in hospitals is real and the risk best managed by encouraging students to remain alert for behavioural changes or other features warning of the potential for aggression [28]. When situations become tense or aggressive, students should withdraw, and seek the assistance of a supervisor [41].

Travel doctors, whilst providing students with valuable expert advice on common travel risk issues (e.g., food safety, vaccine-preventable diseases, road travel, vector-borne diseases), may not be so familiar with some of these clinical occupational risks on electives. Program coordinators should therefore consider providing travel doctors with guidelines specifically relevant to medical electives (Table 4). This information can also be given to students during pre-departure training.

### Theme 5. Pre-departure training programs

Medical schools are increasingly providing comprehensive pre-departure training programs and post-return debriefing sessions for students undertaking medical electives [3, 42–47]. Pre-departure training programs should ideally cover three key areas: a) educational objectives, including expected clinical experiences; b) health and safety, and; c) moral and ethical issues [41, 48–50]. Such programs are likely to be most effective if facilitated by staff with international health experience, and preferably those who are also familiar with emerging concerns about ‘international clinical volunteering’. [9, 47] Programs can also be integrated into mainstream curricula and even be expanded to cover issues such as migrant and refugee health [51, 52]. Collaboration between students undertaking traditional individually organized electives has led to the development of student-directed international health organizations, although the educational and ethical attributes of such organizations may vary somewhat [3, 53, 54]. Students who undertake their electives through volunteer placement organizations typically receive pre-departure information rather than what might be considered worthwhile and multifaceted pre-departure training [9]. The most robust training programs seem to be those that are embedded into long-term, wide-ranging and balanced partnerships with faculty and university level backing [24].

Much of the impetus for providing pre-departure training arises from the desire to improve gaps in student preparedness for international medical electives, including a relative lack of skills training, a need for understanding of novel and challenging cultural and ethical issues, and a lack familiarity with exotic clinical conditions and approaches to healthcare disparity in low-resource areas [55]. Students are also confronted by unfamiliar degrees of suffering and resource disparities while needing substantial clinical supervision, all serving to potentially burden hosts and leave students with lasting feelings of hopelessness and negativity towards the ideals of global health practice [8, 14, 56–58]. Electives in smaller, rural hospitals can be both more closely supervised and ‘hands on’ than those in larger urban teaching hospitals, which may facilitate a smoother transition to the unfamiliar clinical environment [59].

Although some critics believe that pre-departure programs may fall short of being truly socially accountable to the needs and expectations of host communities [60–62], these challenges can be mitigated to some extent by thoughtful scenario-based learning and can be supplemented by opportunities to debrief in groups upon return home. Better still is to abide by an expectation that host institutions will be directly involved in the design (and, ideally, implementation) of such programs [47, 53, 63]. Training programs can be complemented by the use of excellent on-line learning courses in global health ethics, some of which can be undertaken free of charge [64]. Students can be better prepared for their clinical experiences by being directed towards a number of online documents that not only offer insights into the variety of conditions that may be encountered but also highlight the more symptom-based and syndromic approaches to management that are typically employed in lower-income countries [65]. Returned elective students can be enlisted to contribute to pre-departure programs by posing real-life scenarios based on their own experiences. Scenarios can be based on clinical, ethical or health and safety issues. Ultimately, students and their program coordinators should remember that they are first and foremost going on electives to learn under supervision, rather than to be skilled service-providing volunteers. And it may also be timely at this point to remind students of the humanitarian goals of medical practice enshrined (and recently updated) in the World Medical Association’s *Declaration of Geneva* – relevant wherever the setting and whatever the circumstances [66].

### Theme 6. Funding & Finances

International elective placements can be expensive for students. Costs include airfares, accommodation, living expenses, travel insurance, visas, placement fees, HIV prophylaxis and immunisations. Awareness of the extent of the likely costs and means to defray the expenses is a strategic part of effective elective preparation, often up to two years in advance [41, 49, 59].

The medical school sending the students also has expenses running the elective program but these are usually absorbed into the general academic program. However, consideration should be made to making a financial contribution to the host institution that carries the burden of supervision, typically in low resource and high clinical load environments.

### Theme 7. Post-return debriefing and screening

Formal assessment of the elective experience forms an important part of the follow-up process, potentially benefitting program coordinators, students, and host communities. Program coordinators gather useful feedback on elective destinations and on the collective experiences of each cohort. Individual students with problems can be identified and supported. Proper debriefing for students enables them to maintain good health and return to effective study, while reflecting upon and critiquing their cultural capabilities, professional skills, cross cultural communication proficiency, and global citizenship capacity [67]. Whilst the majority of students have positive experiences on their electives, others may return with more mixed feelings that may reflect underlying issues such as physical illness experiences, post-traumatic stress, ‘reverse culture shock’, or general cynicism and nihilism about global health [49]. Post return activities can include group presentation sessions (showcasing elective experiences with peers and academic staff), individual debriefing with elective coordinators, psychological counselling, reflective written assignments, feedback from supervisor reports, medical checks where relevant, and presentations to scholarship funders [68]. All of these activities provide students with an opportunity to reflect on their elective experiences, consolidate learning and understand or contextualize any challenges they have faced. Specialist counselling from faculty members or psychologists who are familiar with global health contexts may also be needed. Longer-term follow-up is less commonly undertaken, however published reviews indicate that international medical electives may lead to a number of lasting benefits, including a desire to continue with similar experiences, development and promotion of similar opportunities for others, greater awareness of the needs of lower-income communities, improved clinical skills, better use of resources, and positive influences on career pathways [25].

Feedback from host institutions on their experiences with individual students and with sending medical schools can also be sought [59, 62]. Host institutions can, in particular, be encouraged to provide constructive feedback beyond a few remarks on the attributes of individual students, commenting on operational issues and reflecting on how future students might be better prepared for the experience.

Students’ physical and mental health should be screened in some form following return from their electives. This may include simple prompts on return, formal screening mental health questionnaires, and offers to have individual consultations. Travel doctors can offer students screening for specific infectious diseases that have been potentially acquired while away, most notably including tuberculosis, HIV infection, malaria, schistosomiasis, and other parasitic infections (including screening for eosinophilia) and sexually transmitted infections. As with pre-departure health consultations, travel doctors may benefit from receiving guidelines specific to post-return screening assessments of elective students (Table 4). Symptomatic or worried students or those with high-risk exposures should be assessed immediately after return. All other students with low likelihood/risk exposures can be conveniently screened 2–3 months following return.

Four other themes emerged and these were deemed to be most relevant to students.

### Theme 8. Clinical routine

While medicine is universal the clinical environments between countries can vary considerably and are starkly different in low resource countries. This means students are exposed to different health systems, clinical approaches, treatments and diseases at a stage in their career where they often have experienced minimal clinical diversity.

Preparation before the elective familiarising with the proposed clinical setting, and required knowledge, attitude and skill set, through contact with the designated supervisor, in the host country is essential [59, 63, 69, 70]. This preparation can be enhanced by home institution pre departure briefings, being informed by previous students who have been to that destination (e.g. through Electives.net and home elective databases) and key text books [71] and government and commercial advisories [72]. There is less difficulty when the placements are longer, at least six weeks, and previous students from the sending university have been there regularly in the previous years, establishing some continuity [73].

### Theme 9. Accommodation & Safety

Safety in a turbulent world with unfamiliar environments requires the student to prepare for known and unknown risks. This includes consideration of travelling companions [27, 39], accommodation [49], ‘street-wise’ behaviour, local and distant travel opportunities, and an understanding of political, social and geographical contexts [6, 23, 28–30, 33, 34, 74]. Preparation can range from simple pre-departure briefings to courses on major humanitarian crises.

### Theme 10. Ethical behaviour and social accountability

Ethical considerations in international medical electives have become a key concern amongst academics, clinicians and students over the last decade [75]. The attitudinal preparation of students has received particular focus [17, 18, 23, 59, 76]. Pre-departure briefings can include guided reflections to help students clarify what they are hoping to achieve and how they can ensure their contributions are positive for themselves and their hosts while promoting ethical behaviour [73]. This can include determining whether the intentions of the volunteer student meet the ideal mindset of being selfless, equitable, and seeking genuine collaboration [8]. Also, being briefed about local customs, cultural variations and ethical traditions relevant to the clinical setting improves outcomes of electives [50]. Assisting the student to reflect on their own worldview, and to develop sensitivity to others worldviews through respectful curiosity and humility will encourage responsible and socially accountable placements. Building on earlier ethical learning in the curriculum and making adjustments to different ethical positions of the hosts can encourage students to adopt a structured approach to cross-cultural ethical issues such as negotiation, privacy, consent, the students clinical role, power, and resource impacts [77].

### Theme 11. Health & Wellbeing

This has largely been covered in the occupational risk and pre-departure briefing themes. Medical students need to avoid the assumption that they are inherently competent in self-care, so therefore specifically prepare for illness in the host country [6]. This includes: actively organising preventative care, knowing what health care is available to them in country, and how to seek assistance and who to inform in the case of illness [23, 25, 49, 78].

## Recommendations

At least one recommendation was made per theme and each recommendation has at least one associated publication. Recommendations for implementation by international medical elective coordinators are organised according to seven of the 11 themes and are listed in Table 2. Adherence to these recommendations is likely to improve the safety and quality of the elective program. Although these recommendations can be applied to other healthcare disciplines our review has largely focussed on coordinators of medical student elective programs.

Recommendations specifically for medical students undertaking electives based on six themes are listed in Table 3. These are practical actions that are the responsibility of medical students, although medical programs should establish guides and resources to promote them.

A separate list of recommendations regarding specific health conditions for travel doctors undertaking occupational health assessment of medical students prior to and returning from international electives can be found in Table 4.

## Conclusions

International medical elective programs are highly valued components of medical school curricula, especially by students, and as such, should be properly resourced, structured and supported. However, there are no internationally accepted or mandated standards for international medical electives program coordinators, or for travel doctors servicing student populations.

We set out to formulate generalizable recommendations by extracting key comprehensive guidelines and supportive literature, analysing them through a systematic qualitative synthesis to produce 11 themes. A limitation of this review is that it relied on expert opinion to form recommendations since there were no existing studies with higher levels of evidence, such as cause-effect research.

Through our critical review of the existing literature we found that, although the experiences of most students are largely positive, many face substantial health and safety risks that could be suitably managed through cooperation between students, medical schools, travel doctors and host institutions. Medical schools must manage their own institutional risks and be respectful and collaborative when seeking partnerships with host institutions. Most importantly, for international medical elective programs to be effective and enriching for all concerned, medical schools and program coordinators should ensure that students’ educational, health and safety, ethical and social accountability needs are met through strategic site visits, comprehensive pre-departure training programs, travel health assessments, and post-return debriefing and health screening sessions.

International medical electives program coordinators should consider implementing the recommendations in this review to promote successful elective experiences for students, their hosts and home institutions. Further studies are critically needed to assess the impact of these recommendations and rigorously compare different interventions.

## Additional file

Additional file 1: Critical Appraisal Checklist for Text and Opinion Papers [13] NB We included any articles that scored 5/6 or 6/6 in this checklist. (DOCX 17 kb)
