# Supplementary material for: Healthy, safe and effective international medical student electives: a systematic review and recommendations for program coordinators
Source: Trop Dis Travel Med Vaccines. 2019 Apr 3;5:4. doi: 10.1186/s40794-019-0081-0 (PMC6448218; doi:10.1186/s40794-019-0081-0)
Supplement: Supplementary file 1 — Critical Appraisal Checklist for Text and Opinion Papers [13] NB We included any articles that scored 5/6 or 6/6 in this checklist. (DOCX 17 kb) [file 40794_2019_81_MOESM1_ESM.docx]

**Additional file 1.** Critical Appraisal Checklist for Text and Opinion Papers^14^

| Reviewer________________________Date _______ |  | | | | |
| --- | --- | --- | --- | --- | --- |
| Author _________________________Year _______ |  |  |  |  |  |
|  |  | Yes | No | Unclear | Not applicable |
| 1. Is the source of the opinion clearly identified? |  | □ | □ | □ | □ |
| 2. Does the source of opinion have standing in the field of expertise? |  | □ | □ | □ | □ |
| 3. Are the interests of the relevant population the central focus of the opinion? |  | □ | □ | □ | □ |
| 4. Is the stated position the result of an analytical process, and is there logic in the opinion expressed? |  | □ | □ | □ | □ |
| 5. Is there reference to the extant literature |  | □ | □ | □ | □ |
| 6. Is any incongruence with the literature/sources logically defended? |  | □ | □ | □ | □ |
| Overall appraisal: Include □ Exclude □ Seek further information □  Comments (Including reason for exclusion): | | | | |  |

NB We included any articles that scored 5/6 or 6/6 in this checklist
